# Supplementary figures and images for: Anaplasma phagocytophilum Transmission Activates Immune Pathways While Repressing Wound Healing in the Skin
Source: Life (Basel). 2022 Nov 24;12(12):1965. doi: 10.3390/life12121965 (PMC9781593; doi:10.3390/life12121965)

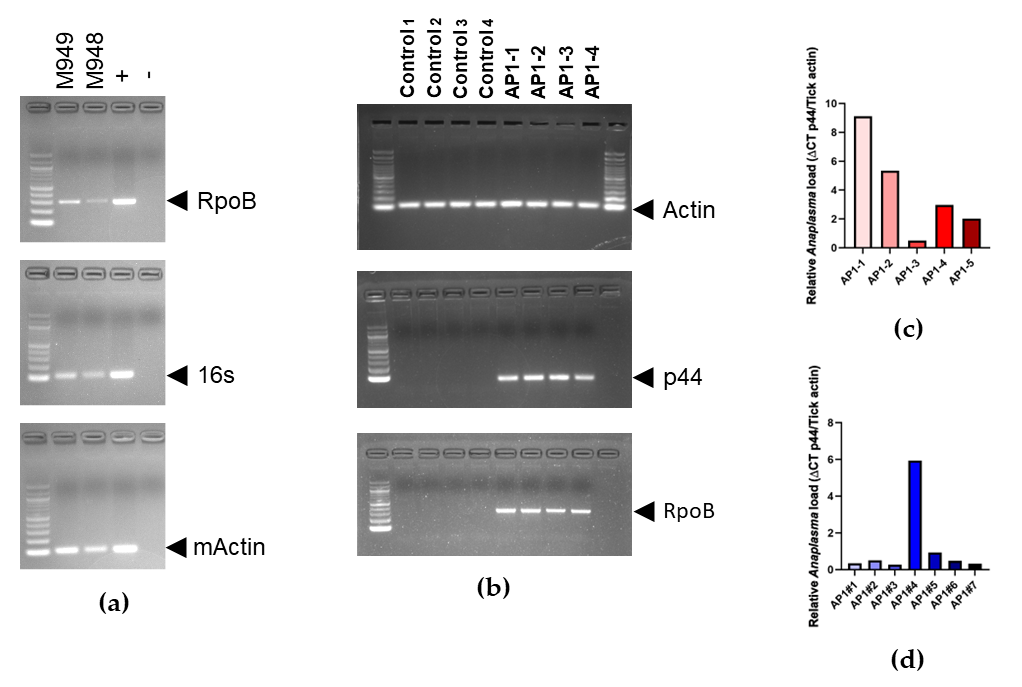

Supplement: Supplementary file 1 [file life-12-01965-s001.zip › Figure S1.tif]

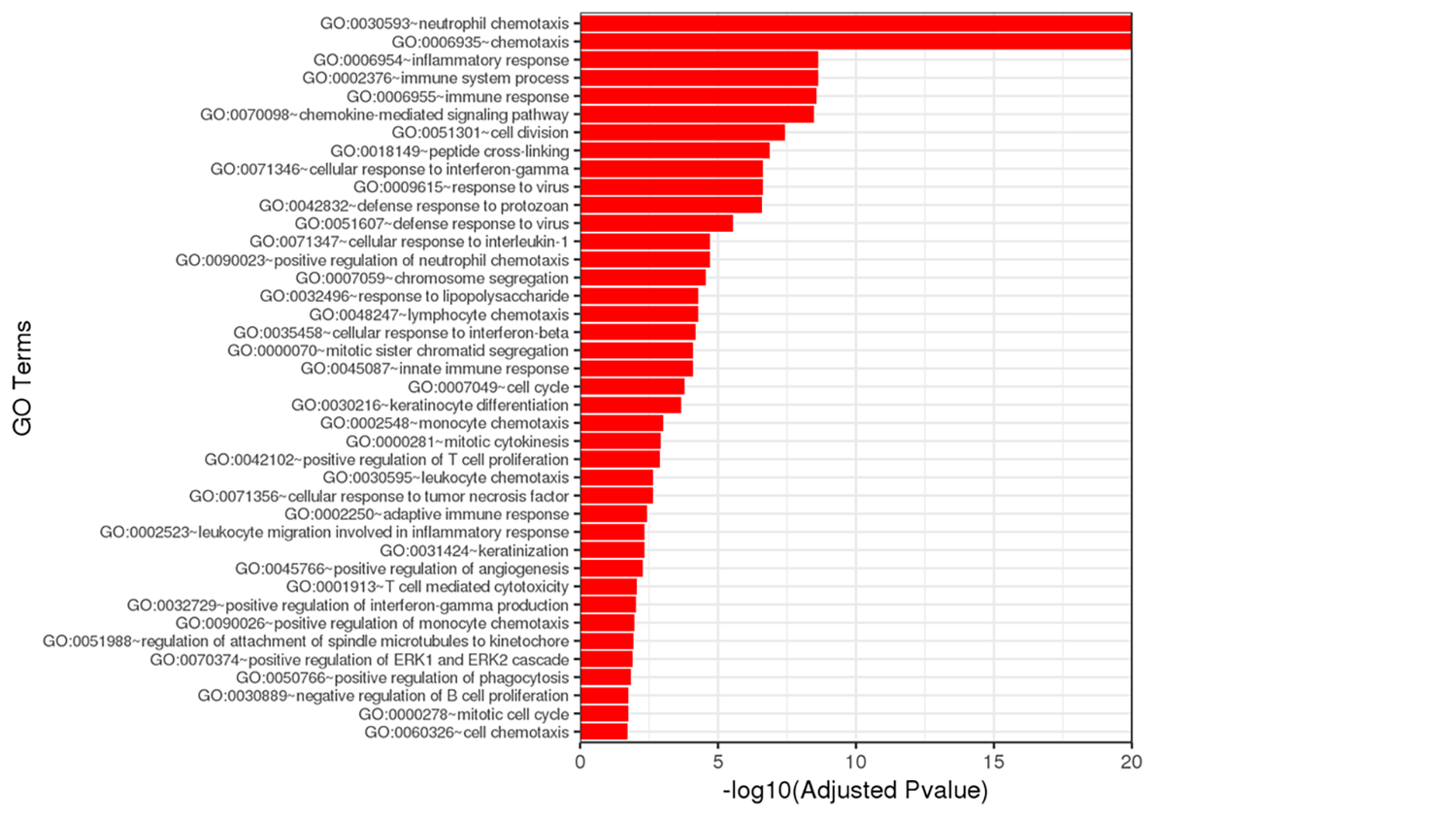

Supplement: Supplementary file 1 [file life-12-01965-s001.zip › Figure S2.tif]

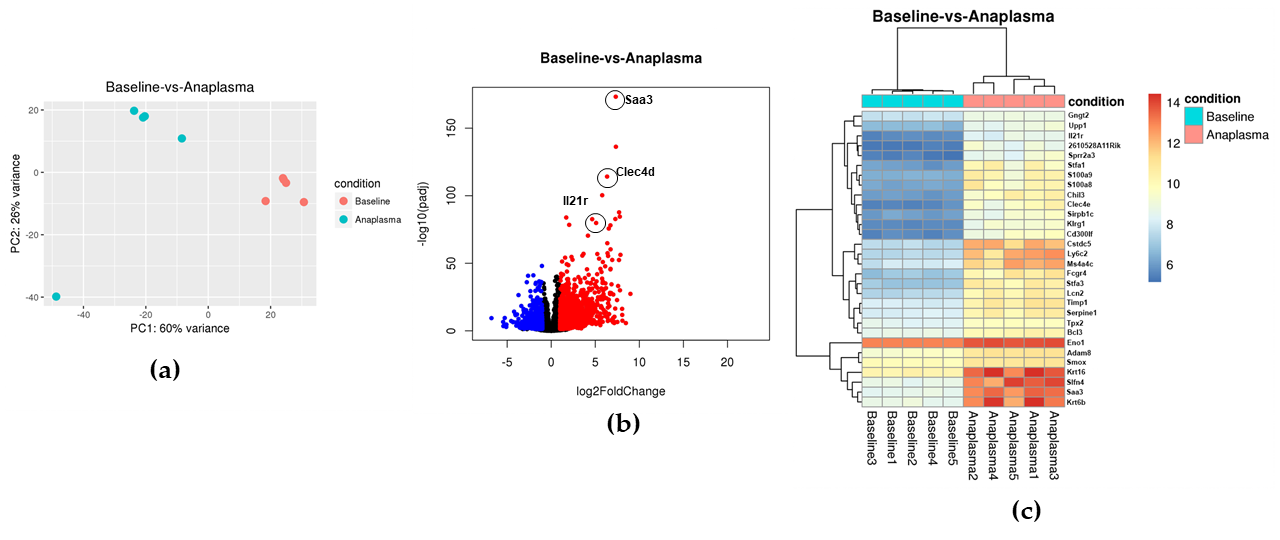

Supplement: Supplementary file 1 [file life-12-01965-s001.zip › Figure S3.tif]

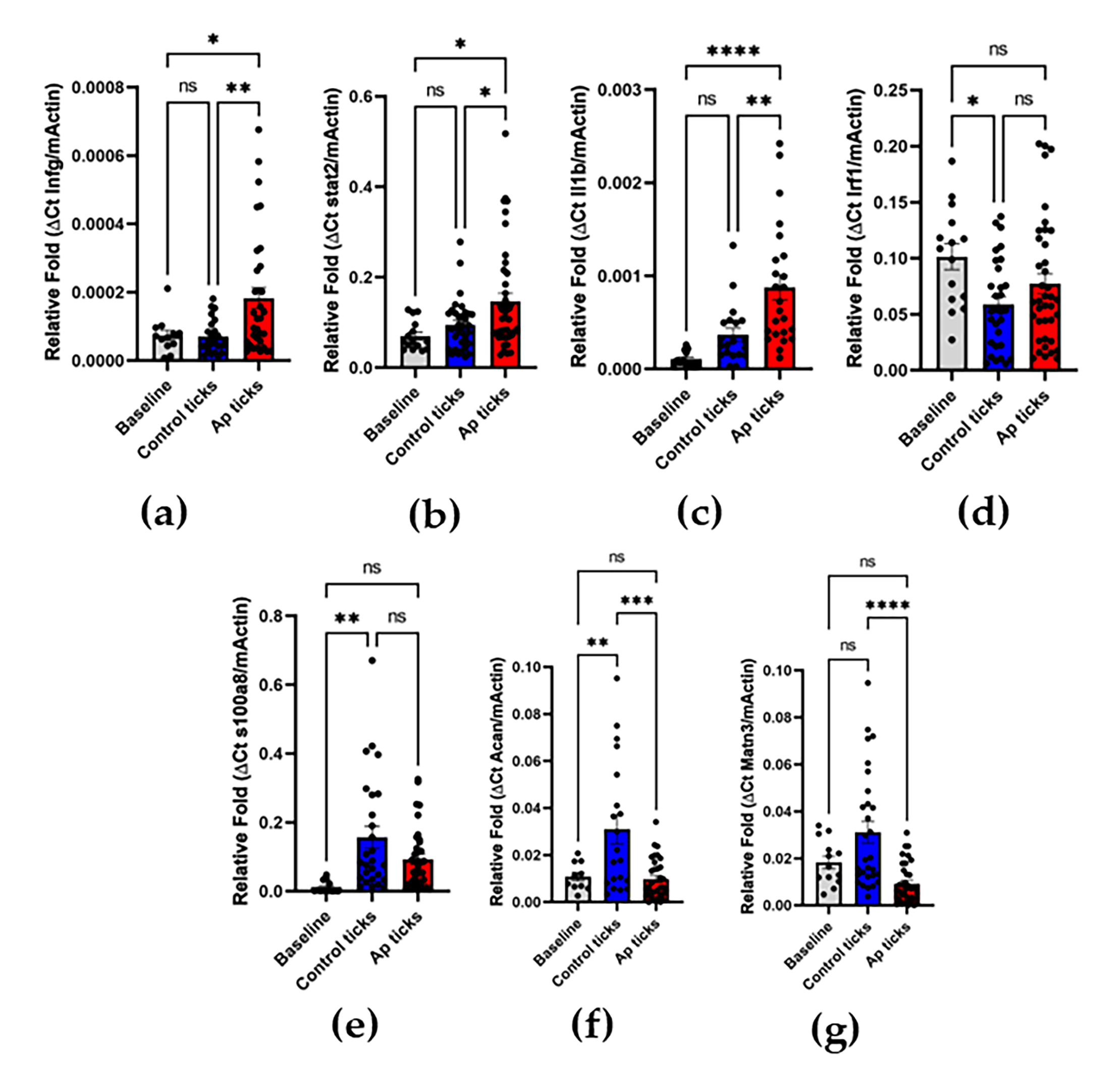

Supplement: Supplementary file 1 [file life-12-01965-s001.zip › Figure S4.tif]

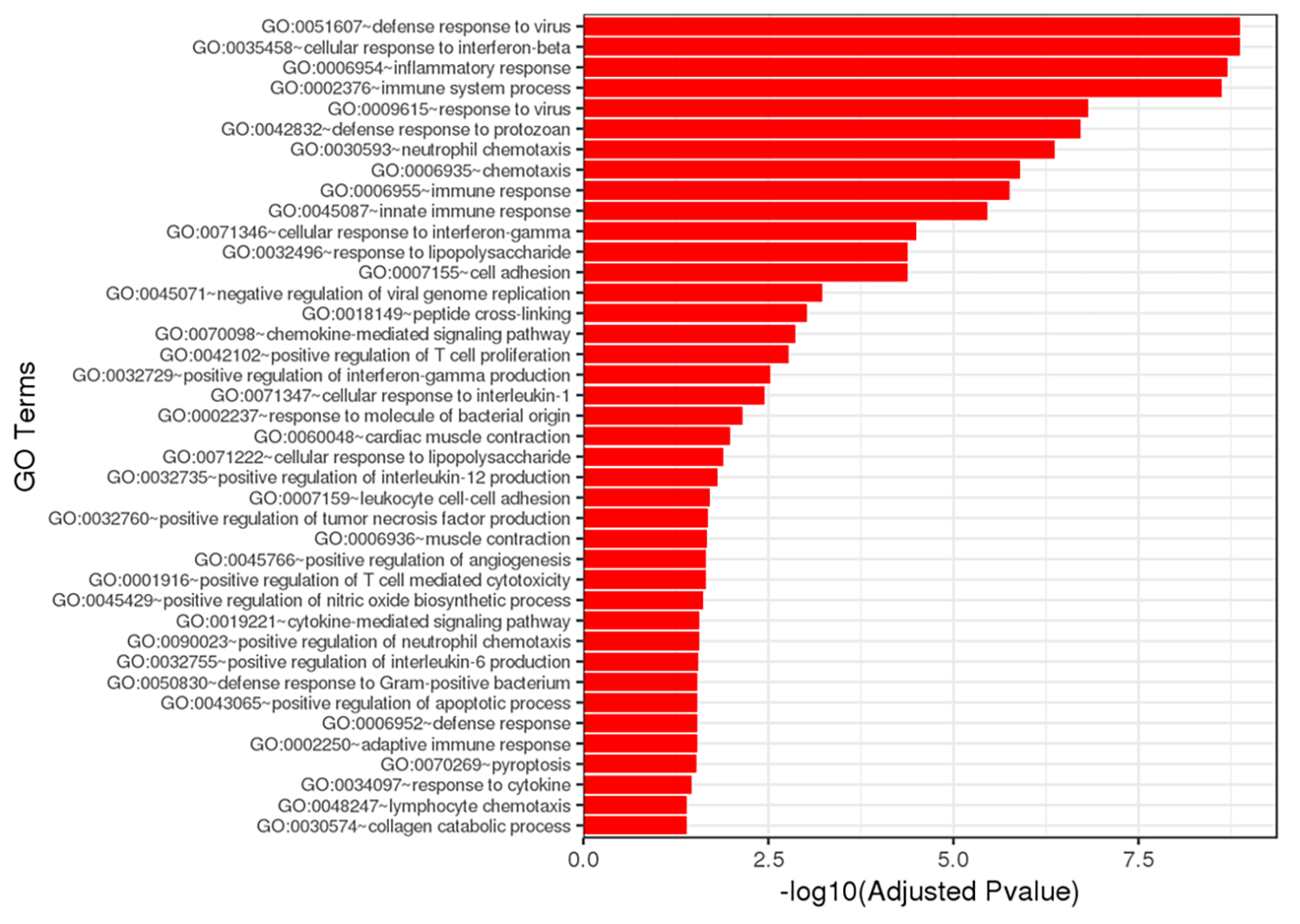

Supplement: Supplementary file 1 [file life-12-01965-s001.zip › Figure S5.tif]
